# Supplementary material for: Exposure Media and Nanoparticle Size Influence on the Fate, Bioaccumulation, and Toxicity of Silver Nanoparticles to Higher Plant Salvinia minima
Source: Molecules. 2021 Apr 16;26(8):2305. doi: 10.3390/molecules26082305 (PMC8071571; doi:10.3390/molecules26082305)
Supplement: Supplementary file 1 [file molecules-26-02305-s001.zip › molecules-1167410-supplementary.pdf]

Exposure media and nanoparticle size influence on the fate, bioaccumulation, and toxicity of silver nanoparticles to higher plant *Salvinia minima*

Supporting information

Melusi Thwala, Stephen J. Klaine, Ndeke Musee*

*Corresponding author: Ndeke Musee; Email: Ndeke.musee@up.ac.za; museen2012@gmail.com

Table S1: The Hoagland's Medium basal salt recipe used in this study.

Chemical	Conc. (mg/L)
$NH_4H_2PO_4$	115.03
H_3BO_3	2.86
$Ca(NO_3)_2$	656.4
$CuSO_4.5H_2O$	0.08
$C_{12}H_{12}Fe_2O_{18}$	5.23

$MnCl_2 \cdot 4H_2O$	1.81
MoO_3	0.016
KNO_3	606.6
$ZnSO_4 . 7H_2O$	0.22
$MgSO_4$	240.76

Table S2: freeze dryer settings used in the experimental setup.

Shelf (°C)	Hold (min)	Vacuum (mTorr)
-40	360	80
-30	360	80
-20	360	80
-10	360	80
0	120	400
20	120	600

Table S3: The achieved recovery rates for Ag obtained from analysis with ICP-MS.

	Sequence Run	QC value	%Recovery			
QC mix + Hg	1st run	2.33	93.29			
Dig 2.5ppb QC	1st run	2.74	109.66			
QC mix + Hg	1st run	2.45	97.91			
Dig 2.5ppb QC	1st run	2.81	112.53			
QC mix + Hg	1st run	2.52	100.62			
Dig 2.5ppb QC	1st run	2.64	105.76			
QC mix + Hg	2nd run	2.28	91.27			
Dig 2.5ppb QC	2nd run	2.81	112.28			
Dig 2.5ppb QC new	2nd run	2.83	113.02			
QC mix + Hg	2nd run	2.49	99.70			
Dig 2.5ppb QC	2nd run	2.69	107.62			
Dig 2.5ppb QC new	2nd run	2.74	109.56			
QC mix + Hg	2nd run	1.95	78.10			
Dig 2.5ppb QC	2nd run	2.45	98.06			
Dig 2.5ppb QC	2nd run	2.65	106.05			
QC mix + Hg	3rd run	2.42	96.73			
Digested 2.5ppb QC	3rd run	2.63	105.12			
QC mix + Hg	3rd run	2.49	99.67			
Digested 2.5ppb QC	3rd run	2.84	113.55			
NB : Digested 2.5ppb QC was digested following the same procedure for the samples						

Table S4: Comparison of whole plant Ag accumulation (µg/mg dry weight) between 10-Ag NPs and 40-Ag NPs under different water chemistries. In brackets are standard deviations, where n = 3. Student's t-test, $p < 0.05$.

	**10-Ag NPs**	**40-Ag NPs**	***p* value**
MHW	0.163 (0.022)	0.120 (0.004)	0.076
NOM	0.154 (0.020)	0.110 (0.020)	0.056
Ca^{2+}	0.193 (0.027)	0.157 (0.0007)	0.149

Table S5: Percentage growth reduction relative to respective controls.

	**10-Ag NPs**	**40-Ag NPs**
MHW	63.08	57.03
NOM	36.28	78.76
Ca^{2+}	1 414.57	1 276.02

Table S6: Results of AgNPs sizes and concentrations under variant exposure media. The 48 h average particle size were obtained with DLS, modal particle size obtained using NTA over 48 h, and NTA was employed to determine particle concentration over 48 h. The given values are the mean ± standard error (n = 3). Differing symbols indicate statistical difference ($p < 0.05$) within a specific AgNPs' size.

			10-AgNPs			40-AgNPs	
Parameter	Time	MHW	Ca^{2+}	NOM	MHW	Ca^{2+}	NOM
DLS size (nm)	48 h	156.85	176.14	257.88*	60.42*	$173.74^{\#}$	107.94^
		(±8.74)	(±17.18)	(±30.24)	(±4.54)	(±13.36)	(±24.48)
NTA size (nm)	0 h	38.33	46.66	68.0	38.33	41.66	39.66
	24 h	(±2.30)	(±4.16)	(±36.42)	(±1.52)	(±1.15)	(±0.57)
	48 h	76.33	88.66	54.33	47.66	52.66	37.00
		(±5.77)	(±3.51)	(±8.50)	(±1.52)	(±4.04)	(±2.00)
		86.33	88.66	64.50	52.00	58.33	38.66
		(±7.50)	(±3.51)	(±6.36)	(±2.64)	(±6.42)	(±0.57)
Particle	0 h	2.12^{8}	4.39^{8}	2.32^{8}	5.86^{8}	5.75^{8}	4.81^{8}
concentration	24 h	(±1.55^{7})	(±1.01^{8})	(±7.21^{6})	(±1.47^{7})	(±9.54^{6})	(±1.31^{7})
(particles/mL)	48 h	1.17^{9}	9.28^{8}	4.68^{8}	7.47^{8}	5.45^{8}	1.48^{9}
		(±4.65^{7})	(±5.51^{6})	(±8.0^{7})	(±5.39^{7})	(±2.51^{7})	(±1.24^{8})
		6.98^{8}	7.54^{8}	2.53^{8}	5.19^{8}	3.94^{8}	1.11^{9}
		(±6.27^{7})	(±1.17^{7})	(±2.12^{6})	(±3.01^{7})	(±7.71^{7})	(±4.13^{7})

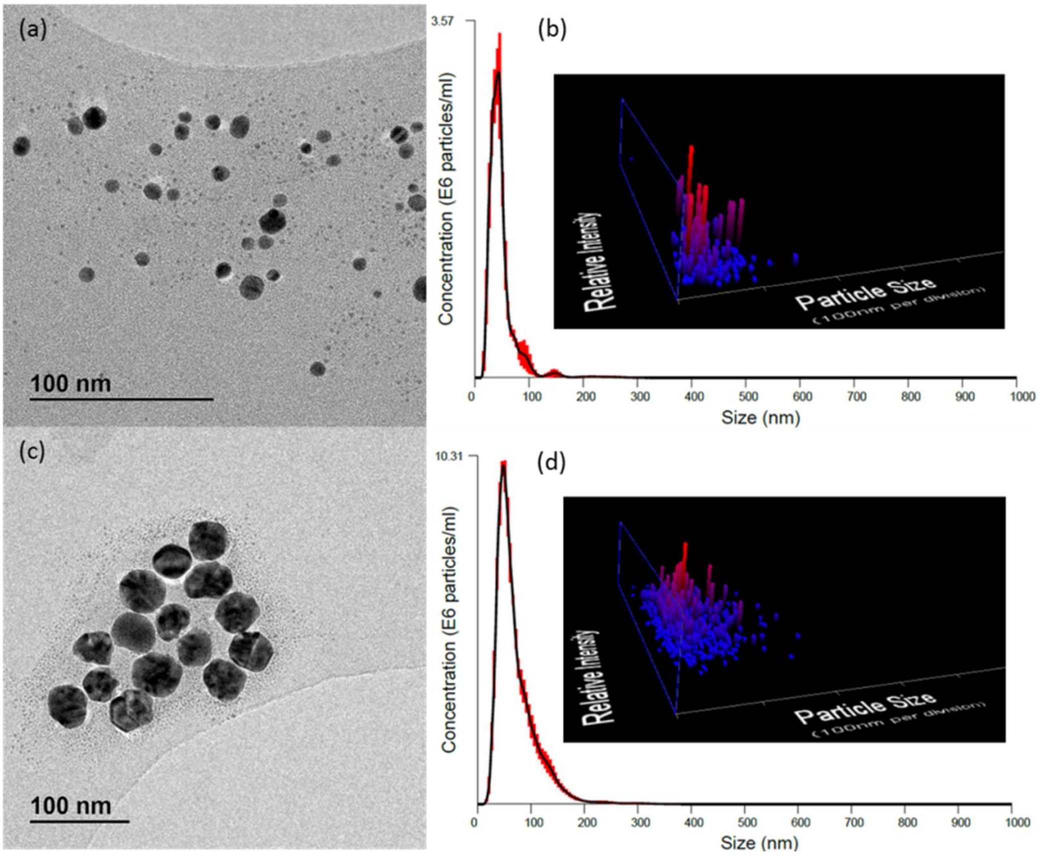

Figure S1: Ag NPs size obtained before testing for 10-Ag NPs with (a) TEM and (b) NTA, and 40-nAg NPs with (c) TEM and (d) NTA. Red bars denote the standard error. Inserts in (c) and (d) illustrate relative Ag NPs size intensities.

MHW 10 nm	Wt%	Wt% Sigma
C	52.01	0.60
O	39.93	0.54
Na	0.25	0.04
Mg	0.87	0.04
Si	0.23	0.02
S	0.99	0.04
Cl	1.47	0.04
K	3.12	0.06
Ca	1.02	0.04
Ag	0.11	0.06
Total	100.00	

Ca 10 nm	Wt%	Wt% Sigma
C	52.79	0.66
O	38.46	0.58
Na	0.21	0.04
Mg	0.52	0.04
Si	0.46	0.03
P	0.79	0.05
S	0.89	0.05
Cl	1.06	0.04
K	2.73	0.06
Ca	1.72	0.05
Cu	0.29	0.07
Ag	0.09	0.07
Total	100.00	

NOM 10 nm	Wt%	Wt% Sigma
C	52.76	0.73
O	39.36	0.66
Mg	0.46	0.04
S	0.77	0.05
Cl	1.49	0.05
K	3.14	0.08
Ca	1.91	0.07
Ag	0.10	0.09
Total	100.00	

MHW 40nm	Wt%	Wt% Sigma
O	64.63	0.52
Na	1.05	0.17
Mg	2.80	0.16
Si	1.07	0.11
P	0.61	0.13
S	3.76	0.15
Cl	5.58	0.16
K	13.39	0.25
Ca	5.79	0.19
Cu	0.90	0.27
Ag	0.42	0.26
Total	100.00	

Ca 40 nm	Wt%	Wt% Sigma
C	52.80	0.66
O	37.23	0.58
Na	0.32	0.05
Mg	0.75	0.04
Si	0.31	0.03
P	0.30	0.04
S	1.29	0.05
Cl	1.23	0.04
K	4.33	0.08
Ca	1.04	0.05
Cu	0.21	0.07
Ag	0.18	0.07
Total	100.00	

NOM 40 nm	Wt%	Wt% Sigma
C	57.19	0.48
O	37.05	0.45
Na	0.27	0.03
Mg	0.38	0.03
Al	0.54	0.03
S	0.64	0.03
Cl	0.62	0.03
K	2.52	0.05
Ca	0.69	0.03
Ag	0.10	0.05
Total	100.00	

Figure S2: The detected elemental analysis for samples exposed to 10-Ag NPs in MWH, Ca, and NOM (top row) and to 40- Ag NPs in MWH, Ca, and NOM (bottom row).

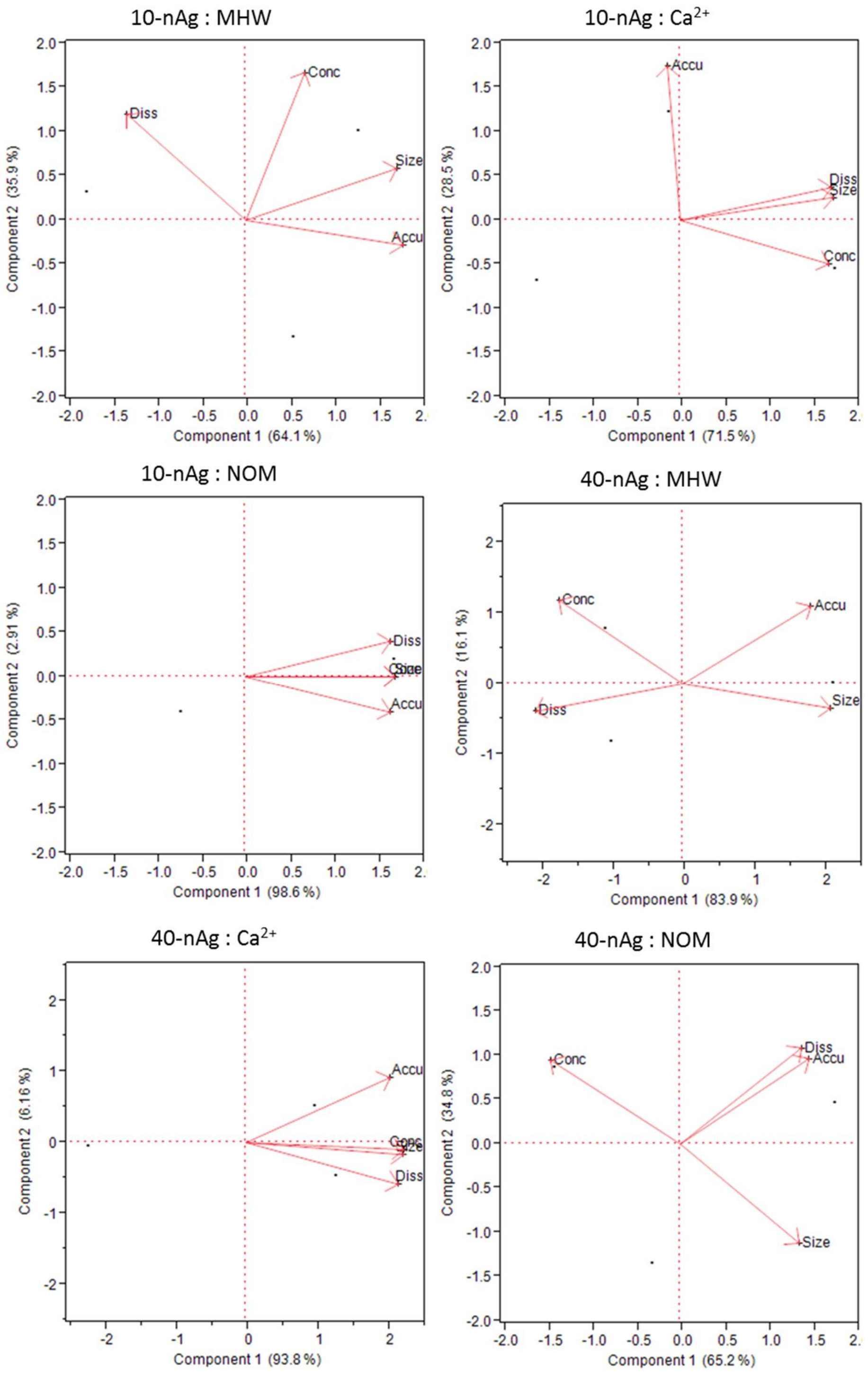

Figure S3: PCA plots illustrating the association of Ag accumulation (accu) to the Ag NPs size (size), dissolution (diss), and Ag NPs concentration (conc) p for 10-Ag NPs and 40-Ag NPs under different water chemistries (MHW, NOM, and Ca^{2+}).

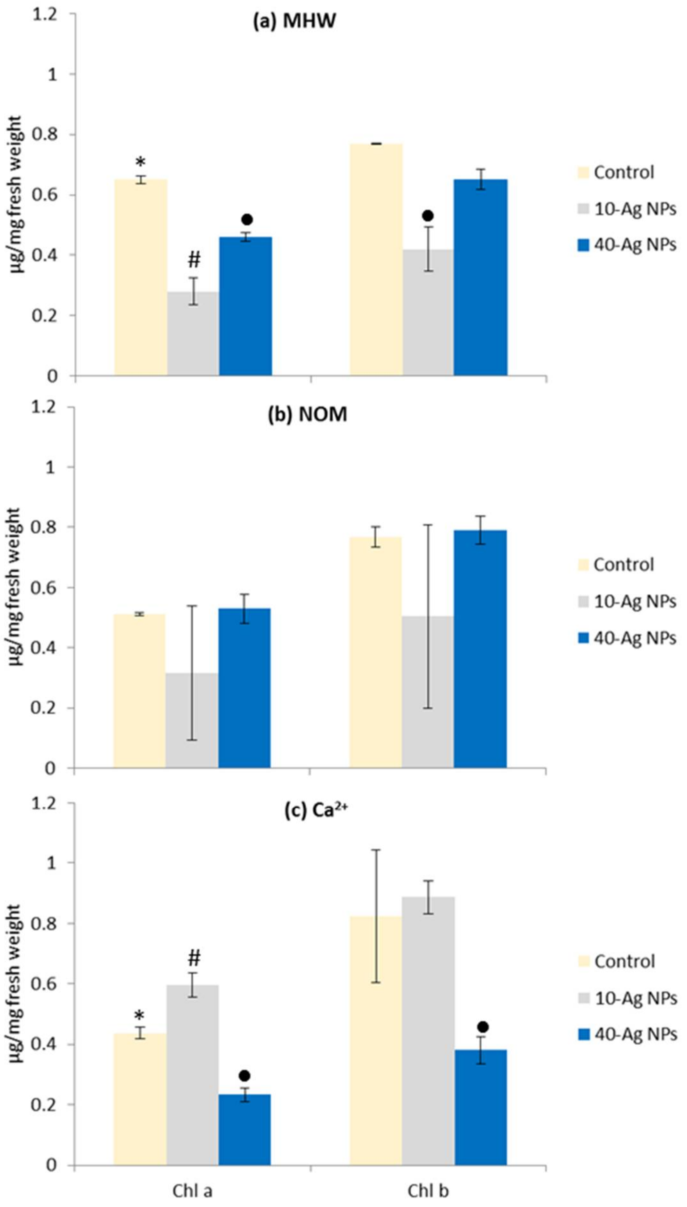

Figure S4: Quantification of chlorophyll pigments Chl *a*, Chl *b* and their ratios in *S. minima* after exposure to 10- and 40-AgNPs for 48 h. Bars denote standard error (n = 3). Differing symbols on top of error bars indicate statistical difference within a specific photosynthetic parameter. Turkey Kramer HSD, $p < 0.05$.
